# Life Cycle of the Water Scorpion, *Laccotrephes japonensis*, in Japanese Rice Fields and a Pond

**DOI:** 10.1673/031.010.4501

**Published:** 2010-05-10

**Authors:** Shin-ya Ohba, P. J. Perez Goodwyn

**Affiliations:** ^1^ Laboratory of Insect Ecology, Graduate School of Environmental Science, Okayama University, Tsushima, Okayama, 700-8530 Japan; ^2^ Laboratory of Insect Ecology, Graduate School of Agriculture, Kyoto University, Kyoto, 606-8152, Japan; ^§^ deceased

**Keywords:** Conservation ecology, habitat utilization, overwintering site, predatory bug

## Abstract

A *Laccotrephes japonensis* (Nepidae: Heteroptera) population was studied based upon mark and recapture censuses in order to elucidate the seasonal pattern of habitat utilization in a rice paddy system including an irrigation pond between April and October, in 2006 and 2007. The seasonal pattern of nymphs and adults did not differ markedly between the rice fields and the pond. Survival rates of *L. japonensis* of all stages did not differ between the rice fields and the pond in 2006, but were lower in 2007 in both habitats. In 2007, however, the survival rate of *L. japonensis* nymphs in the pond was higher than in the rice fields. In rice fields, 36.3% of the overwintering adults were recaptured the following year. On the other hand, the recapture rate after overwintering in the pond was only 6.4%. Migration from the pond to the paddies and vice versa was observed. In summary, the rice fields and the pond may reinforce each other as reproductive and overwintering sites of *L. japonensis*, especially during unfavorable years.

## Introduction

In temporary wetlands void of large fishes, large aquatic heteropterans play a significant role as the major predator of aquatic fauna (Runck & Blinn 1994; Blaustein 1998). Nepidae are reported to feed on a variety of aquatic organisms such as aquatic insects and tadpoles (Menke 1979). In Japan, the water scorpion, *Laccotrephes japonensis* Scott (Nepidae: Heteroptera), is known as large bodied (28–38 mm in body length) and an important predator for both pest control and conservation. *L. japonensis* is an active mosquito larvae predator; however, nymphs of the endangered water bug *Kirkaldyia deyrolli* are also part of the diet (Ohba and Nakasuji 2006, 2007; Ohba 2007). Like many other aquatic insects inhabiting paddy rice systems, *L. japonensis* is declining in some regions in Japan and is designated as a *Red Data List* species in 6 of 47 prefectures (Association of Wildlife Research and EnVision 2007). It is important to study the life cycle of this species in order to obtain fundamental information for more effective management of *L. japonensis* populations in the future.

In recent years, rice fields have attracted concern because of their function as biodiversity conservation areas (Bignal and McCracken 1996; Elphick 2000; Lawler 2001) and as alternative wetlands for many aquatic animals (e.g. Fujioka & Lane 1997; Lane and Fujioka 1998; Maeda and Matsui 1999; Maeda 2001). Rice fields are an important habitat for many aquatic insects, including endangered species in Japan (Saijo 2001; Mukai et al. 2005; Mukai and Ishii 2007). *L. japonensis* is known to prefer lentic and slow-flowing lotic habitats, including paddy rice fields (Ban et al. 1988; Hibi 1994; Hibi et al. 1998), ponds and marshes (Miyamoto 1965), and river margins (Iwasaki 1999). Ban et al. (1988) and Hibi et al. (1998) reported that *L. japonensis* is distributed mainly in the shallow areas of paddy fields. Saijo (2001) reported that *L. japonensis* was seldom found in irrigation ponds and mainly used the paddies for both reproductive and non-reproductive purposes. However, the detailed life cycle and overwintering in rice paddy systems is not well understood. In the present study, mark and recapture censuses were carried out to elucidate the seasonal pattern of habitat utilization by *L. japonensis* in rice paddy fields and an adjacent pond.

## Materials and Methods

### Study sites

Field surveys were conducted in rice fields and at a pond in the western part of Hyogo, central Japan. Rice fields were surrounded by a weed-covered ridge, making a narrow, convenient footpath between adjacent rice fields. The rice fields were initially ploughed and irrigated; then the muddy bottoms were levelled off. Subsequently, the rice fields were filled with 5–15 cm deep water, and the rice saplings were finally transplanted. Water in all rice fields in the study site was maintained from early May to the end of July (irrigation period). In late July, the drainage period started and the water was slowly drained from the field for a few weeks, eventually becoming fully drained, with the ground exposed to the sun. Nevertheless, water in the ditches connecting the rice fields remained at 3–5 cm deep, even during the drainage period. The pattern of rice culture in the site was similar between 2006 and 2007. Censuses were conducted along the ridges around four rice fields and in an adjacent irrigation pond, which was not directly connected. The pond permanently has 100–150 cm of water. The shallowest water strip (from the coast up to 50cm deep) of the irrigation pond was used as the survey area.

### Seasonal activity

To measure the number of *L. japonensis* in the rice fields and in the pond, censuses were conducted from April to October in 2006 and 2007, at intervals of 5–14 days (a total of 25 and 22 occasions during 2006 and 2007, respectively). Censuses were performed by visual observation of *L. japonensis* at night using a flashlight (11,000 1x) from 20:00 to 01:00 h. *L. japonensis* is primarily a nocturnal animal and ambushes prey at the water surface after sunset. Thus, it is much easier to observe at night rather than during the day, and the illumination does not interfere with the behaviour (Ohba and Nakasuji 2006). The observer maintained a constant distance from the water surface (30 cm), and a constant pace (3 m/min walking speed). To maintain sampling consistency, sampling was not conducted during rainy nights. *L. japonensis* adults and nymphs were caught using a 500-µm mesh dipnet (15 cm × 10 cm mouth opening) As a preliminary survey, the number of individuals for both sexes was counted on 6, 24, 30 April 2006, and 7 and 12 May 2006. From the 16 May 2006 survey onwards, newly captured adults were individually identified using colour combination paint dots (Paint Marker®, Mitsubishi) on their thorax. Individual number, generation (overwintered or new-generation adult), and sex were recorded. The prothorax width was measured for the collected specimens. New adults in the population were recognized by the intact wings and/or soft body. After recording, the specimens were released immediately at their point of capture.

The Jolly-Seber method (Jolly 1965; Seber 1965) was applied in order to estimate the number of individuals both in rice fields and in the pond. The total number of *L. japonensis* from all rice fields was pooled together.

### Rice fields and pond as reproductive sites: Comparison of survival rate of nymphs

To estimate the survival rate of *L. japonensis* nymphs, the Kiritani-Nakasuji-Manly method (Kiritani and Nakasuji 1967; Manly 1976) was applied to the frequency of each stage on a series of census occasions. Suppose that the *i*th stage is observed for a time period covered by *n* samples, possibly with a varying time interval between them. The samples are taken at intervals (*h*_1_, *h*_2_ …), and the area under the frequency trend curve is estimated by the trapezoidal rule; for the area *A_i_*: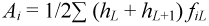 where *f_iL_* = the number of the *i^th^* instar estimated from the samples taken on the *L^th^* occasion, which is at the end of the sampling intervals *h_L_*. There are *h*_*L*+1_ sampling intervals, the last interval extending from the last occasion when the stage was present to the next sampling occasion (when it was found to be absent) (Manly 1976).

Next, the estimated number of each nymphal instar *N_i_* was calculated as: 
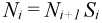
 where *S_i_* denotes the survival rate estimated by the Kiritani-Nakasuji-Manly method for the *i*^th^ nymphal instar. The number of 5^th^ instar was calculated by using the maximum number of new adults estimated by the Jolly-Seber method in each habitat in each year.

The survival analysis with sequential Bonferroni correction (Rice 1989) was used to test for survival curve differences between rice fields and the pond in 2006 and 2007. The Kaplan-Meier method of estimating survival functions and the nonparametric Mantel-Cox log rank test were used. In this analysis, instar and emerged adult were regarded as survival period and censoring, respectively. Statistical significance was set at 0.05. All statistical tests were conducted using JMP version 6.03 (SAS Institute 2005).

### Rice fields and the pond as reproductive sites: Site quality comparison

To evaluate the quality of the sites, the prothorax width of newly emerged adults was compared between specimens caught in the rice fields and specimens caught in the pond from late August to October. A two-way ANOVA was performed with sex and eclosion site (captured site) as the main factors. Log10 transformations for exact values were made to standardize variances and improve normality, if necessary to satisfy the assumptions of the ANOVA model.

**Figure 1.**
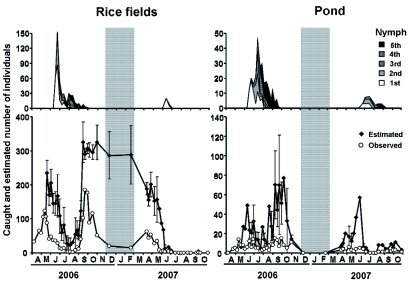
Seasonal changes in the abundance of *Laccotrephes japonensis* at the study site from April 2006 to October 2007. Shaded area indicates winter. Upper graphs for observed number of nymphs, lower ones for adults. For adults, open circles indicate observed number, and filled diamonds denote number estimated by Jolly-Seber method (mean ± SD). High quality figures are available online.

### Overwintering site

To determine whether *L. japonensis* adults were present in the rice fields and in the pond during winter, censuses were conducted on 10 December 2006 and 20 February 2007. Moreover, adults marked from late August to October 2006 (autumn) were followed up from April to May 2007 (spring) in order to estimate the overwintering survival of *L. japonensis.* A multinomial logistic regression analysis was applied to the data of the recaptured specimens in spring (assigning scores of 1 for not captured, 2 for captured in a different site, and 3 caught in the same site) with the recapture data as the dependent variable and site where marked in autumn and sex as the independent variables.

## Results

### Seasonal activity

Occurrence frequency of *L. japonensis* is shown in Figure 1. Adults appeared both in the rice fields and in the pond in April 2006. All adults in the rice fields were found in ditches when water was drained from the rice fields. Mating pairs were found from 16 May to 14 July 2006 (breeding period). First instar nymphs appeared both in the rice fields and in the pond from June to July 2006. From mid-June to August, 2^nd^ and 3^rd^ instar nymphs were first observed, and from June to September, 4th and 5th instar nymphs appeared. Newly emerged adults appeared from late August to October. The occurrence frequency of nymphs did not differ markedly between the rice fields and the pond (Figure 1).

In both the rice fields and the pond, a total of 721 adults were numbered and 438 (61%) were recaptured at least once from May 2006 to October 2007. Out of 157 males and 142 females marked from May to July 2006, only 2 and 1, respectively, were recaptured after April 2007.

Newly emerged adults in 2006 overwintered and then reproduced starting in May 2007, but few nymphs appeared in both the rice fields and the pond. The number of nymphs in 2007 was much lower than in 2006, although the seasonal pattern of occurrence was not different. As a result, in September 2007, only 1 male and 1 female of the new generation were caught in the rice fields, whereas 4 females were found in the pond (Figure 1).

**Figure 2.**
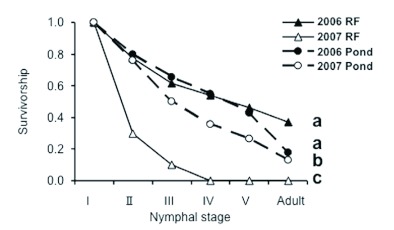
Comparison of survival rates in the rice fields (RF) and in the pond in 2006 and 2007. Different letters at the end of each line denote significant differences (p < 0.05, survival analysis with a sequential Bonferroni test). High quality figures are available online.

### Rice fields and pond as reproductive sites: Comparison of survival rate of nymphs

Survival rates of *L. japonensis* nymphs did not differ between the rice fields and the pond in 2006 (Survival analysis, Mantel-Cox χ^2^ = 0.30, *P* = 0.58; Figure 2). The survival rate in both habitats in 2006 was significantly higher than in 2007 (Mantel-Cox χ^2^ > 26.8, *P* < 0.001 for all combinations). The survival rate in rice fields in 2007 was significantly the lowest (Mantel-Cox χ^2^ > 16.6, *P* < 0.001 for all combinations).

### Rice fields and the pond as reproductive sites: Site quality comparison

Regarding the prothorax width of newly emerged adults, the two-way ANOVA indicated that the effect of sex was significant, but the eclosion site and sex-by-eclosion site interactions were not (sex: *F*_1, 325_ = 605.71, p < 0.001; eclosion site: *F*_1, 325_ = 0.25, p= 0.62; sex-by-eclosion site: *F*_1, 325_ = 0.25, p = 0.62 for log-transformed data). Differences in the prothorax width of newly emerged adults between eclosion sites were not significant for either sex (male: rice fields (*n* = 130) = 7.51 ± 0.03 mm (mean ± SE), pond (*n* = 14) = 7.39 ± 0.209 mm, one-way ANOVA = *F*_1, 142_ = 1.49, p = 0.22; female: rice fields (*n* = 157) = 8.49 ± 0.03 mm; pond (*n* = 28) = 8.42 ± 0.07 mm, one-way ANOVA = *F*_1, 83_ = 0.88, p = 0.35).

### Overwintering site

**Figure 3.**
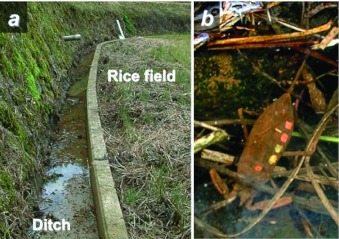
Overwintering site of *Laccotrephes japonensis* in the ditch around (a) rice fields, and (b) ditch. Overwintering adult labeled with color dots on the forewing for individual identification. High quality figures are available online.

There were adult males and females present on the bottom of the ditch connecting the rice fields (8 males and 12 females on 10 December 2006, 3 males and 12 females on 20 February 2007; Figures 1, 3). Estimated number of *L. japonensis* in the rice fields was almost the same between the two surveys (Figure 1). Adults were alone and quiescent on the mud, with their front legs folded up (Figure 3b). However, no adults were found in the pond in any of the two winter surveys.

The results of the recapture experiments in spring 2007 were markedly different from the marked sites in autumn 2006 (the rice fields and the pond) (Logistic regression analysis: Marking site in autumn, *df* = 2, χ^2^ = 22.33, p < 0.001; Sex, *df* = 2, χ^2^ = 2.58, p = 0.275; Marking site in autumn by sex, *df* = 2, χ^2^ = 0.89, p = 0.643). Sex and sex-by-marked-site interaction were not significant effects. Inter-habitat migration was confirmed, both from the paddy field to the pond and vice versa. In the rice fields, of a total of 328 adults numbered in autumn 2006, 119 were recaptured in the rice fields in spring 2007 (36.3%), and 4 adults were recaptured in the pond (1.2%). On the other hand, out of 47 adults marked in the pond in 2006, 3 adults were recaptured in the pond (6.4%) and 4 in the rice fields (8.5%) in spring 2007. Thus, the proportion of recaptured adults in the rice fields was greater than that in the pond.

## Discussion

The results show that *L. japonensis* had a univoltine life cycle in the study site; between mid-May and July, overwintered adults copulate, and the first nymphs appear from June to July. Adults of the new generation appear from late August to October and then overwinter until April of the following year.

### Reproductive site

In 2006, *L. japonensis* nymphs appeared both in the rice fields and in the pond from June to September, as reported by Iwasaki (1999) and Saijo (2001). The occurrence period and survival rate of nymphs were almost the same in the rice fields and the pond (Figures 1, 2). Moreover, the prothorax width of newly emerged adults from the rice fields and from the pond was not different. Thus, the results show the functional equivalency between the rice fields and the pond. The life history pattern is similar to that of *Nepa cinerea* (Southwood and Leston 1959) and *Nepa apiculata* (McPherson and Packauskas 1987). However, the reproductive period was short and clearly discrete, contrary to what Papacek (1989) found.

In 2007, although the reasons are unknown at present, there were few nymphs in the rice fields as well as in the pond (Figure 1). The results suggest an annual fluctuation in the population between 2006 and 2007. The survival rates both in the rice fields and in the pond in 2007 were lower than those in 2006 (Figure 2). However, in 2007 the survival rate in the pond was higher than in the rice fields.

The pond would have played an important role in 2007 as a refuge site. Thus, in the present study site, it may be difficult for *L. japonensis* to subsist exclusively relying on the rice fields.

### Overwintering site

New adults, emerging from late August to October, overwinter in and/or around rice fields and reproduce during the next spring. The recapture rate of overwintered specimens in 2007 was higher in rice fields than in the pond. Iwasaki (1999) studied the life cycle of *L. japonensis* at the river margins of the Yamato-gawa River in Nara, central Japan; however, he could not collect adults from November to March of the next year. In the present study, adults were collected in the ditches around the rice fields during winter (Figure 3). This is the first report on overwintering in water in this species. Species of the closely related genus, *Nepa*, are known to overwinter as adults, also underwater (Southwood and Leston 1959; McPherson and Packauskas 1987; Saulich and Musolin 2007). *L. japonensis* overwinters under the ground in the rice fields, according to Nakayama and Yajima (1985). Individuals not detected in the present study probably overwinter under the ground in the rice fields.

In contrast, few individuals marked in the pond were recaptured in the same habitat after the winter. Although they are related species, *Ranatra chinensis* and *Ranatra unicolor* (Nepidae) overwinter in deeper and permanent water such as ponds (Ban et al. 1988; Hibi et al. 1998); *L. japonensis* may not prefer ponds for overwintering sites.

### Rice fields and irrigation pond joint functioning

This study revealed that both the rice fields and the pond have potential as reproductive and overwintering sites. Nevertheless, the overwintering survival rate in 2006, presumably a favorable year, was higher in rice fields than in the pond, and it was the other way around in 2007. Thus, the pond may play a role as a refuge site in comparison with the rice fields, especially when an unfavorable annual fluctuation occurs, because of the higher survival rate and the active migration. The migration from the pond to the paddies would be expected, as the Nepidae are considered “passive migrants” (Kanyukova 2006), providing there was a water connection between both habitats. In this case, however, the active migration from the paddies to the pond and vice versa was confirmed. The migration method is unknown, but an adult was found walking from one rice field to another during May 2006 (unpublished data). Most adults probably walk in order to migrate before overwintering.

In this study site, poorly drained ditches were suitable to cover the whole life cycle of *L. japonensis* even during the drainage period. However, this is not the case in many rice paddy systems, where the drainage from August onwards would have a large impact on the population dynamics of this species. Land consolidation, which is the conversion of poorly drained rice fields into well-drained dry rice fields using a below-ground drainage system, tillage in winter, and winter cropping will reduce the overwintering survival of this species, as was reported for the belostomatid bug, *Appasus major* (Mukai and Ishii 2007).

In conclusion, the rice fields and irrigation pond reinforced each other as reproductive and overwintering shelter sites of *L. japonensis.*
